# Mobile Mindfulness Intervention on an Acute Psychiatric Unit: Feasibility and Acceptability Study

**DOI:** 10.2196/mental.7717

**Published:** 2017-08-21

**Authors:** Lisa A Mistler, Dror Ben-Zeev, Elizabeth Carpenter-Song, Mary F Brunette, Matthew J Friedman

**Affiliations:** ^1^ Dartmouth-Hitchcock Medical Center Department of Psychiatry Geisel School of Medicine at Dartmouth Concord, NH United States; ^2^ Director, mHealth for Mental Health Program Department of Psychiatry and Behavioral Sciences University of Washington Seattle, WA United States; ^3^ Department of Anthropology Dartmouth College Hanover, NH United States; ^4^ Dartmouth-Hitchcock Medical Center Department of Psychiatry Geisel School of Medicine at Dartmouth Lebanon, NH United States

**Keywords:** mindfulness, meditation, mHealth, psychiatry, mobile phone, aggression, violence, schizophrenia, bipolar disorder, psychotic disorders

## Abstract

**Background:**

Aggression and violence on acute psychiatric inpatient units is extensive and leads to negative sequelae for staff and patients. With increasingly acute inpatient milieus due to shorter lengths of stay, inpatient staff is limited in training and time to be able to provide treatments. Mobile technology provides a new platform for offering treatment on such units, but it has not been tested for feasibility or usability in this particular setting.

**Objective:**

The aim of this study was to examine the feasibility, usability, and acceptability of a brief mindfulness meditation mobile phone app intended to reduce anger and aggression in acute psychiatric inpatients with schizophrenia, schizoaffective disorder, or bipolar disorder, and a history of violence.

**Methods:**

Participants were recruited between November 1, 2015 and June 1, 2016. A total of 13 inpatients at an acute care state hospital carried mobile phones for 1 week and were asked to try a commercially available mindfulness app called Headspace. The participants completed a usability questionnaire and engaged in a qualitative interview upon completion of the 7 days. In addition, measures of mindfulness, state and trait anger, and cognitive ability were administered before and after the intervention.

**Results:**

Of the 13 enrolled participants, 10 used the app for the 7 days of the study and completed all measures. Two additional participants used the app for fewer than 7 days and completed all measures. All participants found the app to be engaging and easy to use. Most (10/12, 83%) felt comfortable using Headspace and 83% (10/12) would recommend it to others. All participants made some effort to try the app, with 6 participants (6/12, 50%) completing the first 10 10-minute “foundation” guided meditations.

**Conclusions:**

This is the first known study of the use of a commercially available app as an intervention on acute psychiatric inpatient units. Acutely ill psychiatric inpatients at a state hospital found the Headspace app easy to use, were able to complete a series of meditations, and felt the app helped with anxiety, sleep, and boredom on the unit. There were no instances of an increase in psychotic symptoms reported and there were no episodes of aggression or violence noted in the record.

## Introduction

Despite the lack of consistency in accurate measurement, it is clear that violence on psychiatric inpatient units is a significant problem. In a recent meta-analysis of 35 studies involving more than 23,000 acutely ill psychiatric inpatients in the United States, Iozzino [1] reported that the pooled proportion of patients engaging in at least one act of violence while hospitalized was 17%. The hypothesized and measured costs to staff and patients are manifold. The most obvious cost to staff is physical injury [2,3]. One state hospital in the United States recently reported that of the 425 assaults of staff in 2014, 394 resulted in a workplace injury, and from 2012 to 2014, the hospital logged 5600 cumulative days of missed work [4]. Additional problems related to inpatient violence include decreased workplace morale, job dissatisfaction, increased staff turnover [3,5], increased use of coercive practices [2,6], decreased patient satisfaction and engagement with care [7], plus increased readmission rates [8].

Agitation, impulsivity, and disorganized thinking are cited as common causes of, or contributors to, patient aggression [9,10]. There are effective cognitive behavioral therapies (CBT) for addressing aggression and violence [11,12]. However, these rely on an intact cognitive capacity, which is often impaired in those with serious mental illness (SMI). A number of other potential problems with CBT for anger and aggression in people with SMI have been raised, including the need for introspective ability and self-awareness, as well as a perception that there is a problem with anger or aggression [11].

In several small studies, mindfulness-based therapies (MBT) have successfully reduced impulsivity and agitation, which underpin aggression and violence [11,13-16]. Mindfulness interventions have been proposed as alternatives to CBT for use with people who have cognitive impairment or disorganized thinking, as mindfulness improves emotion regulation without requiring the cognitive restructuring emphasized in CBT [17,18]. Mindfulness-based interventions teach an alternative “aware and observing” approach related to sensations, thoughts, and feelings so as to promote *acceptance of* rather than *reacting to* everyday life, especially during high-stress situations. Importantly, MBT have been used safely and successfully to improve quality of life in persons with SMI, particularly in those with treatment-resistant hallucinations [18-23], as well as to address aggression in a small number of individuals with SMI [24-26]. Mindfulness-based stress reduction has been shown to reduce contributors to aggression, such as impulsivity, stress and anxiety, negative mood states, and depression, among individuals with SMI and active psychotic symptoms [19].

In mental health care, mobile technologies such as mobile phones are being used more frequently to induce individuals to engage in increased self-monitoring and treatment outside of the provider’s office [27-29]. Mobile technologies are portable and computationally powerful, and therefore have the potential to provide the right dose of the right intervention at the right time of individualized evidence-based mental health treatment. Mobile health approaches are just beginning to be tested in people with SMI; however, early work has demonstrated that this treatment approach is feasible and acceptable to psychiatric outpatients [30-32] and inpatients [33-35]. These pioneering studies demonstrate that people with SMI can successfully use mobile technology as long as the technology is user-friendly, intuitive, and engaging, and the treatment model includes self-management components that are independent of clinician engagement [36].

Using a mindfulness app on mobile phones to address violence on acute psychiatric units addresses several needs in the field. Although effective cognitive behavioral interventions have been developed to address this problem, they are difficult, if not impossible, to provide on an acute inpatient unit due to increased symptom acuity, decreased lengths of stay, and lack of staff time and training. Using an *existing*, *commercially available* mindfulness app in this context has other potential advantages. From a research standpoint, there is far less cost and time needed to develop the initial intervention. Additionally, this app can be used by inpatients whenever and wherever they need an intervention, providing another potential coping skill that can be used even after discharge and independent of the research project. Unlike apps specifically designed for research studies, this commercially available and relatively inexpensive app would be readily available to discharged individuals.

Demonstrating that a mindfulness mobile phone app can provide engaging treatments that inpatients with SMI can access when they want is innovative because it combines 2 techniques, mindfulness and mobile technology, each of which has been independently and successfully used in this population, but not in this combination. This study sought to determine the feasibility of utilizing a mindfulness mobile phone app by acutely ill psychiatric inpatients with schizophrenia, schizoaffective disorder, and bipolar disorder. It also sought to determine how patients felt about using the mindfulness app.

## Methods

The Committee for Protection of Human Subjects at Dartmouth College and New Hampshire Department of Health and Human Services approved the study. Participants provided informed consent. Study participants were recruited primarily by daily screening of the hospital census by the research assistant (RA) between November 1, 2015 and June 1, 2016.

### Participants

Patients diagnosed with schizophrenia, schizoaffective disorder, or bipolar disorder, aged 18-65 years, with a recent (within the 6 months before admission) history of aggression or violence were eligible for the study. Participants were screened for reading at least at a 6th grade level. Exclusion criteria included having a hearing, vision, or major motor difficulty that made it impossible for them to use a mobile phone. People with guardians were excluded. Inpatients were compensated US $50 for completing the 7-day study.

### Study Flow

A total of 50 individuals were approached, 27 declined to participate (54%) and 13 enrolled (26%). The average length of stay at the hospital is quite short and the level of symptom acuity is high; therefore, many potential participants were too ill to complete the competency screener when approached initially and those who were able to participate were often close to their day of discharge and were unlikely to stay the full 7 days required for the study. One participant was discharged unexpectedly before completing the 7 days of the intervention and we were unable to meet with him in time to complete the follow-up assessment before he left the hospital, thus the final sample included 12 participants. Of these 12, 10 used the app for the 7 days of the study and completed all measures. Two additional participants used the app for fewer than 7 days and completed all measures. Data from all 12 participants are included in this paper.

### Procedures and Measures

Trained research staff met with interested individuals and described the study in detail. If the candidate continued to be interested, the RA reviewed the consent form and administered a short competency screener to assess whether they understood what the study entailed. The RA administered the word knowledge section of the WRAT-4 (Wide Range Achievement Test 4) [37] to evaluate whether candidates were at least at a 6th grade reading level. After giving their informed consent, the enrolled participants completed the baseline assessments and were provided with a mobile phone with phone functions and camera disabled (in order to reduce the likelihood of confidentiality violations) to use for the 7 days of the study. At the first visit, baseline demographic measures and study measures of mindfulness, anger, and cognition were also conducted.

Participants were taught how to use Headspace, a mobile phone app that aims to teach beginners the basic concepts of mindfulness through simple guided meditations.

The Headspace app has an initial “Take 10” program that consists of 10 10-minute meditations guided by a male voice identified as “Andy.” Completing the initial 10 guided meditations unlocks access to more materials, including a “Series Library,” and 3 “Foundation Level” packs that make up a total of 30 sessions. The 10 sessions in the Foundation Level 1 are all 10 minutes long. The 10 sessions in Foundation Level 2 can be completed as either 10- or 15-minute sessions. The 10 sessions in Foundation Level 3 can be completed in 10-, 15-, or 20-minute sessions. Levels 2 and 3 are not required, but encouraged. Headspace data collected include number of sessions completed, a breakdown of the type of sessions, the average session length, and total time spent in meditating. The RA instructed participants to follow the daily mindfulness exercises feature of the “Take 10” program for at least 10 minutes a day over 7 days.

### Quantitative Data Collection

At the end of the study, participants completed a rating scale adapted from a previous work by Ben-Zeev et al [32] that assessed acceptability and usability of the app. The study augmented these quantitative findings with qualitative methods to examine study participants’ experiences using the Headspace app.

### Qualitative Data Collection

The investigators created a semistructured interview based on previous work done by one of the authors (DBZ) using an interview topic guide that followed the “funnel structure” described by Krueger and Casey [38]. Questions in the interview were designed to elicit study participants’ attitudes toward and positive and negative experiences using the app. Questions also addressed how participants used the app on the unit, what barriers hindered use, and what facilitated use. The RA then asked each participant a series of semistructured interview questions about their experiences with the app. Interview questions focused on users’ views of the amount of time they spent engaged in mindfulness practice, or the “dose” of the intervention, whether they felt the intervention was interesting, engaging, or useful, and what negative effects, if any, they encountered. Finally, participants were asked for their reflections on how the experience and/or the app might be improved.

### Statistical Analyses

Simple statistics were used to describe the study sample. The audiotaped interviews were transcribed, and the Principal Investigator and RA read all transcripts and listened to the interviews to identify prominent issues, ideas, and perspectives that emerged from the data. Transcripts were reviewed by multiple analysts to develop an initial codebook, based on categories from the interview guide as well as content present in participants’ responses. The transcripts were then coded to find and label relevant text passages with the appropriate qualitative codes. Related codes were then grouped into the broader categories presented in the results. Through an iterative process of discussing emergent codes and re-reading transcripts, they reached a consensus on the main findings [39]. Five main themes emerged from this study: (1) usability of the app and equipment, (2) therapeutic applications, (3) barriers to use, (4) suggestions for program adaptation, and (5) endorsement of use for peers.

## Results

The study group included 12 participants (males, N=10, 83%; whites, N=11, 92%; mean age 33.4 [SD 10.7]; mean total years of education 12.6 [SD 2.6]). The majority of participants were unemployed (9/12, 75%). All participants had prior psychiatric hospitalizations, 5 (5/12, 42%) within the past year. Twelve participants completed the pre- and posttest assessments, including the usability and acceptability questionnaire and the qualitative interview. The most common diagnosis of the participants was schizoaffective disorder (6/12, 50%). There were equal numbers of participants with diagnoses of bipolar disorder (3/12, 25%) and schizophrenia (3/12, 25%).

**Table 1:** Participant usability and acceptability questionnaire.

Statement	Strongly disagree n (%)	Disagree n (%)	Neutral n (%)	Agree n (%)	Strongly agree n (%)
I would use the app often	1 (8)	1 (8)	2 (17)	6 (50)	2 (17)
It was too complicated	4 (33)	8 (67)	0 (0)	0 (0)	0 (0)
It was easy to use	0 (0)	0 (0)	1 (8)	4 (33)	7 (58)
I felt confident using it	0 (0)	0 (0)	1 (8)	4 (33)	7 (58)
I felt comfortable using it	0 (0)	0 (0)	2 (17)	3 (25)	7 (58)
It was easy to learn	0 (0)	0 (0)	0 (0)	5 (42)	7 (58)
The info was easy to understand	0 (0)	0 (0)	1 (8)	4 (33)	7 (58)
I could see the screen	0 (0)	0 (0)	2 (17)	2 (17)	8 (67)
I had enough meditation time	0 (0)	2 (17)	4 (33)	2 (17)	4 (33)
I did not have enough meditation time	6 (50)	3 (25)	2 (17)	1 (8)	0 (0)
I did not like the voice	6 (50)	5 (42)	1 (8)	0 (0)	0 (0)
I did like the voice	0 (0)	0 (0)	3 (25)	5 (42)	4 (33)
The app made my voices worse	10 (83)	2 (17)	0 (0)	0 (0)	0 (0)
The app made me more anxious	7 (58)	5 (42)	0 (0)	0 (0)	0 (0)
The app made me less anxious	0 (0)	0 (0)	1 (8)	9 (75)	2 (17)
The app helped me focus	0 (0)	0 (0)	3 (25)	6 (50)	3 (25)
The app did not help me focus	5 (42)	7 (58)	0 (0)	0 (0)	0 (0)
The app helped me manage symptoms	0 (0)	1 (8)	3 (25)	5 (42)	3 (25)
The app made me more upset	7 (58)	4 (33)	1 (8)	0 (0)	0 (0)
The app functions the way I want	0 (0)	0 (0)	1 (8)	8 (67)	3 (25)
I would use the app in the future	0 (0)	2 (17)	2 (17)	5 (42)	3 (25)
The app was fun to use	0 (0)	1 (8)	2 (17)	5 (42)	4 (33)
I would recommend the app to others	0 (0)	0 (0)	2 (17)	6 (50)	4 (33)
I was comfortable with the info collected on the app	0 (0)	1 (8)	1 (8)	7 (58)	3 (25)
I was worried about the privacy of my info	2 (17)	5 (42)	0 (0)	5 (42)	0 (0)
I found it easy to keep the phone with me	1 (8)	3 (25)	0 (0)	5 (42)	3 (25)

### Quantitative Results

The quantitative results from the usability and acceptability questionnaire are shown in Table 1. Participants were asked to rate each statement on a scale from 1 (strongly disagree) to 5 (strongly agree), with 3 rated as neutral.

### Qualitative Results

#### Usability

Uniformly, all participants endorsed that both the phone and the app were user-friendly, regardless of age. Elements that contributed to usability included that the app was easy to learn, the information provided was easy to understand, and the screen was clear. This is important in light of published results that people with SMI may require special adaptations to mHealth interventions to accommodate cognitive impairment and neurological deficits produced by medications [40,41]. Only 1 participant reported difficulty in navigating from one section of the app to another, as in below:

My overall impressions were that the app is very streamlined. The interface is very easy to use, even for someone who’s potentially like an older user, someone over the age of 50 or 60. Um, for me it was very simple and almost mindless to navigate into the app.

In general, people felt it was relatively easy to keep the mobile phone with them on the unit. Anecdotally, before initiation of the study, unit staff had previously hypothesized that participants would be losing, misplacing, or even throwing the phones away; however, it turned out that over time, staff became invested in the project to the extent that they were reminding participants to charge their phones and were prompting them to use the app when they became agitated. Of note, no phones were lost and only 1 was broken during this study of acutely ill participants with SMI. When asked how the phone was broken, the participant stated that he sat on the phone when it was in his pocket, and thus the screen cracked. There was no other collateral information available regarding the phone breaking, including nothing in staff notes about an aggressive act using the phone.

#### Therapeutic Applications

Most of the participants reported using the skills they learned using the Headspace program; for example, some noted having increased awareness of their body and surroundings and that they were better able to focus. Participants commented on using the app to help fall asleep in particular. They also said they used the breathing techniques to help control anger, decrease anxiety, and improve mood. While measures of state and trait anger did not change significantly after the intervention, participant references to the app helping control their anger is promising for future studies:

Helped me dealt with the day’s events. If I had too much trouble during the day, then I would just go to my room and uh, do some coloring or think about what Headspace did for me in the morning and concentrate on something entirely than what’s going on out in the dayroom.

Interestingly, several people indicated that the app helped with boredom or getting through the day during their involuntary stay at the hospital. This is consistent with other findings that the treatment environment is a major contributor to aggression, especially when there is a lack of activity or variation in activity [42,43]. We know from the literature that periods of inactivity and boredom are consistent with an increased likelihood of aggression and violence [43]; therefore, this study reinforces the idea that interventions that alleviate boredom could conceivably be used to reduce aggression in future research, as in the following cases:

Headspace helped keep the days filled with positive activities.

I was looking forward to the 10 minute session in the morning, because I would get up and say, “Geez, you know, I got something to do this morning, I got, I got my uh, my Headspace to look forward to.”

For all but one person, active symptoms were not identified as a barrier to use of the app. Several participants endorsed the idea that a mindfulness intervention would lead to an increased awareness of symptoms while simultaneously allowing the participant to “let go” of emotional attachment to symptoms, as follows:

I think it actually highlighted some of my symptoms that are, not in a bad way, it’s just...I am more aware of, um, intrusive thoughts and things like that, you know, um, so, in a good way, it helped me to learn a little bit more about how my brain works and some of the faults that I have...

The...way he described meditation..., as your mind starts to wander, it’s almost like he knew...“All right, his mind’s wandering now,” like...and if your mind wanders, bring it back to your breathing...

#### Barriers to Use

The most common response to questions about barriers to use was that the hospital units were too noisy and the atmosphere was not private enough to engage in the meditation app, as in below:

Um, I, I really couldn’t find a really good place to not hear anything. I...because in, in this mental institution kind of thing, you can’t really find a good place to like sit down and actually relax. but I-, I could sometimes but when I could it helped out a lot.

Well, thing was, was because I had my roommate, and I had people in the hallway constantly, so it was kinda hard to always be on point with it.

Another barrier to enjoyment of use was having less control over the app. Unfortunately, the lack of a wireless connection in the hospital led to a more cumbersome process for moving from one Headspace “level” to another once the next level was unlocked; participants reported that needing to wait for the RA to unlock the next level for them was somewhat frustrating. Several participants did not like the advertisements for purchasing Headspace embedded in the program. Only 1 participant reported technological difficulties in trying to use the program on one occasion. Only 1 person indicated that he felt his illness may have interfered with his ability to participate fully in the study:

Uh, just because, uh, just because of my own personal, uh, problems that I have with thinking clearly. Uh, I feel like it’s, uh, just not as effective on me as it may be on a lot of other people.

Although 5 participants agreed with the statement “I was worried about the privacy of my information” on the Usability and Acceptability Questionnaire, this was not a recurring theme in the qualitative interviews. In fact, when asked if there were any other issues he wanted to bring up at the end of the qualitative interview, 1 participant stated:

Just please try to keep my identity and private information private, ’cause we live in a world where there is no real privacy.

Despite the reported obstacles, including the noise and crowding on the units, most of the participants managed to find ways to use the Headspace app. This level of participation in an activity that required some concentration and commitment from study participants is remarkable, given that participants were involuntarily admitted individuals with symptomatic SMI.

#### Program Adaptation Suggestions

Participants had several creative ideas for adapting the intervention for future use. A number of participants suggested making the program more interactive with other participants, which is a feature normally available for Headspace; however, as mentioned previously, the hospital does not currently have WiFi capability and thus this feature was not an option. In addition, participants recommended having other activities available on the phone, such as games. One participant stated that the equipment was somewhat obtrusive, with a large phone and “big giant headphones:”

You might be able to scale it down to a point where you could fit it onto something portable, way portable, way more portable.

Another common theme for adaptation recommendations was to increase the flexibility of the app itself to include more choices, such as what type of voice would be heard during the guided meditations, as well as to have a bigger variety of meditations that were aimed at particular problems that patients were seeking to address, as in below:

Um add, um, a woman speaker, like um, like a uh-, like you can, like right in the beginning, do you want to listen to a woman or do you want to listen to a man?

Um, if you had certain categories to choose from, what mood setting and what situation you might have gone through before the session

One person eloquently described the potential benefits of having readily accessible feedback on participation in the app:

Like if there was an opportunity to go and take a little survey before and after a meditation or before and after a course I think that would, uh, I think that can make a difference, too. Seeing all those little bar graphs, those little digital bar graphs for some people it’s just like wow the, uh, the numerics of everything, the metrics or everything really amaze a lot of people out there in the digital world, so I think that would, with keeping the s-, with the graphical user interface staying similar I think, uh, if th-, if those metrics were added people would just, they would, it would make Headspace pop really.

The recommendation for increased interaction with peers and for having games and other features on the phones is consistent with the hypothesis that patients have a lot of “down time” on the units and could conceivably be bored.

#### Endorsement of Use for Peers

Most participants said they would recommend Headspace to others. They commented on their own use of the breathing techniques, increased ability to focus, and having an increased awareness of their surroundings. Several participants endorsed having had philosophies consistent with mindfulness before engaging in the study, as follows:

Whatever thoughts are happening, accept ’em, you know, he said that a lot. You just kind of accept what’s going on, but you do it in a nice, relaxed way.

Participants felt it was refreshing to be offered something they could use by themselves at their own pace that did not involve medications or formal face-to-face interactions.

## Discussion

### Principal Findings

Out of 5 psychiatric inpatients, 1 engages in at least one act of violence during their stay [1], resulting in negative effects on staff and patients. There is a critical need for effective interventions that reduce aggression and violence and can be delivered to acutely ill psychiatric inpatients within a brief inpatient stay. With increasingly acute inpatient milieus due to shorter lengths of stay, inpatient staff has limited training and time to be able to provide such treatments. Mobile technology provides a new platform for offering treatment on such units, but it has not been tested for feasibility or usability in this particular setting. The objective of this study was to determine feasibility, usability, and acceptability of a brief mindfulness meditation mobile phone app intended to reduce anger and aggression in acute psychiatric inpatients with schizophrenia, schizoaffective disorder, or bipolar disorder, and a history of violence.

To our knowledge, this study reports on the first deployment of a commercially available app as an intervention on acute psychiatric inpatient units. Overall, participants with active affective and psychotic symptoms were able to understand and use a mobile mindfulness app during their admission to an inpatient psychiatric unit. There was no evidence of worsening of symptoms or induction of psychotic symptoms as a consequence of app use. Qualitative data indicate that the majority of participants liked the app for many reasons, including that it gave them “something to do” and seemed to provide a sense of mastery or control over something during an involuntary hospitalization.

Of note, many participants indicated that he app helped them relax enough to sleep better. Use of nonpharmacologic approaches to address sleep, anxiety, and even agitation could potentially reduce polypharmacy attributable to pro re nata (*prn*) medications on inpatient units. *prn* medications are given to between 70% and 90% of all psychiatric inpatients [44], and are hypothesized to lead to increased morbidity due to increased likelihood of drug interactions, dependence or misuse, and polypharmacy [45]. The literature supports that insomnia and anxiety are two of the three most common reasons for distribution of *prn* medications [46]. Some findings indicate that patients that use *prn* s end up feeling a loss of autonomy or feeling coerced [47]. Using skills instead of medications to address anxiety, insomnia, aggression, and mood is safer and more likely to lead to better results over the long term [48]. Encouraging patients to use such alternatives gives them more of a sense of empowerment and agency in managing their own symptoms, participating in their own care [46], and encourages a more collaborative relationship with providers [49]. Many psychiatric (and nonpsychiatric) patients understandably want to use “natural” means for therapy, reducing their exposure to potentially noxious side effects of, and interactions between, psychotropic medications

Several patients noted the benefit of having “something to do” on the unit. Boredom is reported as a precursor to aggression and violence on inpatient psychiatric units by patients themselves in qualitative studies [43]. Ostensibly, psychiatric inpatient units are the most intensive treatment modality in mental health care. Yet, patients report that they are bored on psychiatric units, with a limited range and quality of available activities and difficulty in getting time to talk with staff [43]. In one UK study, 40% of psychiatric inpatients reported not participating in social or recreational activity and 30% in no structured activity at all during their admissions [50]. With shorter hospital stays and more acutely ill patients, staff activity involving direct patient care tends to be more “putting out fires” involving a minority of severely ill patients on the unit, rather than engaging in proactive care for all patients [51,52]. mHealth interventions offer an alternative mode of treatment that may also alleviate the tedium of being on an inpatient unit.

### Limitations

This study has several limitations including a small sample size and brief data collection period (driven by the short average length of hospitalizations). There is research supporting that groups of at least five are sufficient to identify most usability problems [53,54]. However, studying larger groups would likely provide more information [55]. There is also the potential for biased responses in the qualitative interviews and the Usability and Acceptability Questionnaires, as they were conducted by the study team’s RA rather than by an independent interviewer, due to funding limitations. Future studies should include a qualitative interviewer who is not part of the study team in order to minimize the likelihood of such bias. One would generally expect that over time, participants would have improved functioning due to use of medications and other therapies. However, it was clear from the interviews that several participants had ongoing thought disorganization and hallucinations.

There were some difficulties related to hospital policy regarding mobile phones; charging phones required that staff help participants, as there is a prohibition on having cords on the unit for safety reasons. Not having access to a wireless connection meant that there was no ability to use what could be the most powerful features of Headspace, interaction with others using the app, keeping track of one’s progress, and competing with others.

This work supports the idea that it is feasible to offer acutely psychiatrically ill inpatients a commercially available mindfulness meditation app and that the patients are able to use the app with few difficulties. The next step is to study the potential relationship between use of the app and inpatient aggression and violence. Future work will involve a more rigorous evaluation of a mindfulness intervention and its effects on aggression and violence on psychiatric units.

### Conclusions

This is the first known study of the use of a commercially available app as an intervention on acute psychiatric inpatient units. As such, acutely ill psychiatric inpatients with SMI were able to use the app, navigating through it without much reported difficulty, and described using the mindfulness techniques to help with sleep and anxiety while on the unit. This is an example of an mHealth intervention that could potentially deliver individualized treatment when it is needed in an environment where the staff may be too busy to work one on one with the hospitalized person. Further study is warranted based on these findings.

## Abbreviations

CBT: cognitive behavioral therapies
MBT: mindfulness-based therapies
prn: pro re nata
RA: research assistant
SMI: serious mental illness
WRAT-4: Wide Range Achievement Test 4
